# Sex‐specific manifestation of genetic risk for attention deficit hyperactivity disorder in the general population

**DOI:** 10.1111/jcpp.12874

**Published:** 2018-02-16

**Authors:** Joanna Martin, Mark J. Taylor, Mina Rydell, Lucy Riglin, Olga Eyre, Yi Lu, Sebastian Lundström, Henrik Larsson, Anita Thapar, Paul Lichtenstein

**Affiliations:** ^1^ Department of Medical Epidemiology & Biostatistics Karolinska Institutet Stockholm Sweden; ^2^ MRC Centre for Neuropsychiatric Genetics and Genomics Cardiff University Cardiff UK; ^3^ School of Medical Sciences Örebro University Örebro Sweden; ^4^ Institute of Neuroscience and Physiology Gillberg Neuropsychiatry Centre Centre for Ethics Law and Mental Health University of Gothenburg Gothenburg Sweden

**Keywords:** ADHD, anxiety, depression, genetics, CATSS, ALSPAC

## Abstract

**Background:**

Attention deficit hyperactivity disorder (ADHD) is more commonly diagnosed in males than in females. A growing body of research suggests that females with ADHD might be underdiagnosed or receive alternative diagnoses, such as anxiety or depression. Other lines of reasoning suggest that females might be protected from developing ADHD, requiring a higher burden of genetic risk to manifest the disorder.

**Methods:**

We tested these two hypotheses, using common variant genetic data from two population‐based cohorts. First, we tested whether females and males diagnosed with anxiety or depression differ in terms of their genetic risk for ADHD, assessed as polygenic risk scores (PRS). Second, we tested whether females and males with ADHD differed in ADHD genetic risk burden. We used three different diagnostic definitions: registry‐based clinical diagnoses, screening‐based research diagnoses and algorithm‐based research diagnoses, to investigate possible referral biases.

**Results:**

In individuals with a registry‐based clinical diagnosis of anxiety or depression, females had higher ADHD PRS than males [OR(CI) = 1.39 (1.12–1.73)] but there was no sex difference for screening‐based [OR(CI) = 1.15 (0.94–1.42)] or algorithm‐based [OR(CI) = 1.04 (0.89–1.21)] diagnoses. There was also no sex difference in ADHD PRS in individuals with ADHD diagnoses that were registry‐based [OR(CI) = 1.04 (0.84–1.30)], screening‐based [OR(CI) = 0.96 (0.85–1.08)] or algorithm‐based [OR(CI) = 1.15 (0.78–1.68)].

**Conclusions:**

This study provides genetic evidence that ADHD risk may be more likely to manifest or be diagnosed as anxiety or depression in females than in males. Contrary to some earlier studies, the results do not support increased ADHD genetic risk in females with ADHD as compared to affected males.

## Introduction

Attention deficit hyperactivity disorder (ADHD) is a common and impairing disorder, with a prevalence of approximately 5% in childhood (Polanczyk, de Lima, Horta, Biederman, & Rohde, 2007). ADHD is a neurodevelopmental disorder, characterised by onset in childhood, male bias and high levels of comorbidity with other neurodevelopmental (e.g. autism spectrum disorder) and psychiatric (e.g. anxiety, depression) phenotypes (Thapar, Cooper, & Rutter, 2017). Although childhood ADHD is 2–9 times more prevalent in males (Nussbaum, 2012; Polanczyk et al., 2007), it is unclear how and why this sex bias is observed.

Attention deficit hyperactivity disorder diagnostic criteria are potentially more geared towards identifying symptoms typical in males, given that field trials for establishing diagnostic criteria were based on a sample including only 21% females (Lahey et al., 1994). As the male bias is more pronounced in clinically ascertained samples than in population‐based samples (Nussbaum, 2012), it is likely that females are routinely underdiagnosed with ADHD and that phenotypic differences, referral or diagnostic biases (e.g. professional awareness or diagnostic substitution) play a role. In community samples, females meeting ADHD criteria generally present with less severe ADHD symptom profiles and fewer comorbid problems than affected males; in contrast, clinical studies find few sex differences, although females show more impairment from inattentive symptoms (Gaub & Carlson, 1997; Gershon, 2002). This pattern of less distinctive sex differences in clinically ascertained compared to nonclinical samples further suggests that ADHD may be underdiagnosed in females compared to males.

Literature reviews have generally concluded that females are more likely than males to present with inattentive, rather than hyperactive‐impulsive symptoms, as well as comorbid anxiety/depression; conversely, the combined subtype of ADHD is more common in males, who more frequently present with comorbid conduct‐related behaviours (Quinn, 2008; Quinn & Madhoo, 2014; Rucklidge, 2010; Staller & Faraone, 2006; Willcutt, 2012). Thus, research suggests that the presentation and associated comorbidities of ADHD may differ by sex.

It has been suggested that ADHD may be underdiagnosed in females due to the less overt nature of inattentive symptoms, as well as possible diagnostic overshadowing from anxiety, eating or depressive disorders (Quinn, 2008; Quinn & Madhoo, 2014). Furthermore, the male‐to‐female ratio for ADHD is much reduced in adults (nearer to 1:1), possibly indicating a delay in females receiving ADHD diagnoses (Faraone et al., 2015). The pattern of symptomatic sex differences in diagnosed females and males from clinically referred and population childhood samples is consistent with a greater symptom threshold requirement for ADHD referral of females. This suggests that only the most severely affected females are referred for clinical assessment and diagnosed with ADHD in childhood. Less severely affected females, who nonetheless manifest ADHD traits above the diagnostic threshold and experience impairment, might instead receive alternative diagnoses (Quinn, 2008).

To date, no study has directly examined the contribution of genetic risk factors to putative sex‐specific diagnostic biases. ADHD is highly heritable, with heritability estimated at ~70%–80% (Faraone et al., 2015). A recent ADHD genome‐wide association study (GWAS) meta‐analysis identified the first robust single nucleotide polymorphisms (SNPs) associated with ADHD and estimated a SNP‐based heritability of approximately 22% (Demontis et al., 2017). Sex‐stratified genetic analyses have shown that the SNP‐based genetic correlation for ADHD in males and females is very high (close to 1), suggesting that largely the same common genetic risk variants contribute to ADHD in both sexes (Martin et al., 2017). Molecular studies have also shown that common risk variants are shared between ADHD and major depressive disorder (genetic correlation (*r*_g_) = .26), depressive symptoms (*r*_g _= .45) and anxiety disorders (*r*_g _= .40) (Anttila et al., 2017; Demontis et al., 2017). Twin studies have similarly reported shared genetic risk between ADHD and internalising problems (additive genetic correlation (*r*_A_) = .45), depression (*r*_A _= .67–.77), affective problems (*r*_A _= .34–.48) and anxiety (*r*_A _= .45–.58) (Chen et al., 2016; Cole, Ball, Martin, Scourfield, & Mcguffin, 2009; Michelini, Eley, Gregory, & McAdams, 2015; Rydell, Taylor, & Larsson, 2017). However unlike ADHD, there is a female excess in depression and anxiety (Craske et al., 2017; Weissman et al., 1996). These observations raise the possibility that ADHD genetic risk manifests differently in females and males in the general population, for example as anxiety or depression in females.

Although referral and diagnostic biases are potentially an important contributing factor to observed sex differences in ADHD prevalence, they are unlikely to fully explain this difference. Whole population‐based screens for ADHD consistently find higher mean ADHD symptoms in males (Larsson, Anckarsater, Råstam, Chang, & Lichtenstein, 2011; Martin, Hamshere, Stergiakouli, O'Donovan, & Thapar, 2014; Nussbaum, 2012), suggesting that other factors also contribute to the sex bias in prevalence.

One alternative hypothesis is that females are in some way resilient to developing ADHD, a theory known as the ‘female protective effect’ (Taylor et al., 2016). Indeed, several family studies have reported that first‐degree relatives of affected females are at increased risk of ADHD, compared to relatives of affected males (Martin et al., 2017; Rhee & Waldman, 2004; Smalley et al., 2000; Taylor et al., 2016). This is indirect evidence for a higher burden of genetic risk in females diagnosed with ADHD and their relatives. However the evidence is mixed, with two studies not finding this effect (Chen et al., 2016; Faraone, 2000). Also, although molecular genetic studies (one clinical and one population‐based) found a higher burden of common risk variants in females with ADHD (Hamshere et al., 2013; Martin et al., 2014), a much larger clinical ADHD study reported no sex difference in ADHD common variant burden (Martin et al., 2017). Additional replication of genetic burden analysis is needed in population‐based samples.

The aim of this study was to test two related hypotheses for the sex‐biased prevalence of ADHD using Swedish and British population‐based cohort studies. First, we hypothesised that ADHD genetic risk is more strongly associated with anxiety or depression diagnoses in females than males. Second, we hypothesised that females with ADHD have an increased burden of ADHD genetic risk as compared to diagnosed males. To test these hypotheses, we examined whether in individuals who had anxiety, depression or ADHD diagnoses, females had a higher burden of ADHD genetic risk than males, using polygenic risk scores.

## Method

### Description of samples and measures

#### CATSS

The Child and Adolescent Twin Study in Sweden (CATSS) is a population study of all twin children born in Sweden since July 1992 (Anckarsäter et al., 2012). Since 2004, the cohort is being recruited when twins turn 9 years old. They are assessed at ages 9, 15, 18 and 24 years using parent telephone interviews and postal/Internet questionnaires. Initially, families with 12‐year‐old twins were also recruited. Parents gave informed consent to participate in the study on behalf of themselves and their children. The study was approved by the Karolinska Institutet Ethical Review Board in Stockholm.

DNA samples (from saliva) have been collected since 2008 from the participants at initial recruitment to the study. Samples were genotyped using the Illumina PsychChip genotyping chip. Standard quality control (QC) and imputation procedures were performed in the sample; for details see (Brikell et al., 2017). *N* = 13,576 samples and *N* = 6,981,993 imputed SNPs passed QC. Children with brain damage/injury or chromosomal abnormalities were excluded. This left *N* = 13,472 children included in analyses.

Information on ADHD, anxiety and depression was available from two sources: registry‐based clinical diagnoses as well as parent‐ and twin‐rated screening measures (hereafter referred to as ‘screening‐based research diagnoses’). Notably, the registry‐based diagnoses are subject to potential referral and diagnostic biases, whereas the screening‐based diagnoses are less likely to be affected by sex‐specific biases.

Data on clinical diagnoses given by specialist services were obtained from the Swedish National Patient Register (NPR) through linkage using each individual's personal identification number. The NPR contains information on inpatient psychiatric care from 1987 and specialist outpatient consultations from 2001. It includes best‐estimate specialist diagnoses according to ICD‐10 (International Classification of Diseases version‐10) codes (WHO, 1993). ICD codes (including subheadings) were obtained for the following diagnoses: ADHD/hyperkinetic disorder (F90), social anxiety and phobias (F40), generalised anxiety and panic disorders (F41), separation anxiety and other childhood‐onset anxiety disorders (F93), single and recurrent major depressive disorders (F32‐F34). Diagnoses were collapsed into four binary variables: ADHD, any anxiety disorder, any depressive disorder, and any anxiety or depressive disorder. At the end of follow‐up (2014), the individuals in this cohort study were aged between 9 and 22 years old. Individuals diagnosed with depression prior to age 13 were excluded from analyses, as the typical onset of depression is during adolescence and adulthood (Weissman et al., 1996).

As part of the CATSS research study, parents completed the Autism‐Tics, ADHD and Other Comorbidities inventory (A‐TAC) (Hansson et al., 2005) over the telephone, when the children were aged 9 or 12 years old. ADHD was assessed using two modules: inattention and hyperactivity/impulsivity, corresponding to the 18 DSM items for childhood ADHD, and one additional item related to boredom, scored on a 3‐point scale (total possible range = 0–19). This ADHD measure has been reported to have good construct validity, with an area under the curve of 0.90, sensitivity of 0.92 and specificity of 0.73 for detecting a broad screening proxy of ADHD (score ≥ 6) (Larson et al., 2010). Study individuals were also screened for anxiety and depression at age 18. Parents rated symptoms of anxiety (6 items) and depression (15 items) in their children, using the Adult Behavior Checklist (ABCL) (Achenbach, Dumenci, & Rescorla, 2003) and the twins self‐reported on anxiety, using the Screen for Child Anxiety Related Emotional Disorders (SCARED) (Birmaher et al., 1997), and on depression, using the Center for Epidemiologic Studies Depression Scale (CES‐D) (Radloff, 1977). Total scores on each instrument were dichotomised so that the top 5% of responders were classed as being affected, to ensure that the most severely affected individuals were captured, while maximising statistical power.

#### ALSPAC

The Avon Longitudinal Study of Parents and Children (ALSPAC) is a large, well‐characterised longitudinal study (Boyd et al., 2013; Fraser et al., 2013). ALSPAC originally recruited *N* = 14,541 pregnant women resident in Avon, England, with expected delivery dates between 1 April 1991, and 31 December 1992. An additional 713 eligible children were enrolled after age 7, resulting in a total sample of *N* = 14,701 children alive at age 1 year. The study website (http://www.bris.ac.uk/alspac/researchers/data-access/data-dictionary/) contains details of all available data through a fully searchable data dictionary. Ethics approval was obtained from the ALSPAC Ethics and Law Committee and Local Research Ethics Committees.

Children from triplet and quadruplet births were excluded and only one child from twin births was included in genotyping. *N* = 9,912 ALSPAC children were genotyped using the Illumina HumanHap550 quad SNP genotyping platform. Detailed QC procedures have been published previously (Martin et al., 2014). After QC, genome‐wide data were available for *N* = 8,215 children and *N* = 1,813,169 imputed SNPs.

Unlike in CATSS, information on ADHD, anxiety and depression was not available from national registers, only from parent‐ and child‐rated screening measures. In ALSPAC, these screening measures also included information on impairment and were used to derive algorithm‐based research diagnoses.

Psychiatric disorders were assessed at multiple time points using a semistructured interview, the Development and Well‐Being Assessment (DAWBA) (Goodman, Ford, Richards, Gatward, & Meltzer, 2000), which assesses symptoms and impairment. Parents completed the DAWBA ADHD, anxiety and depression sections when their children were aged 7, 10, 13 and 15 years (N.B. only ADHD section completed at 15) and adolescents completed the anxiety and depression sections at age 15 years. Computer‐generated DAWBA band variables predict the probability of a psychiatric disorder being present (Goodman, Heiervang, Collishaw, & Goodman, 2011). Following recommendations, >50% probability of diagnosis was classed as ‘diagnosis likely present’ and lower levels as ‘diagnosis likely absent’. These bands have been well‐validated in community samples of British and Norwegian children, showing excellent agreement with clinician‐rated diagnoses (Goodman et al., 2011). Participants also completed the CIS‐R (Clinical Interview Schedule‐Revised) (Lewis, Pelosi, Araya, & Dunn, 1992), a computerised interview to assess anxiety and depression, at age 18 years. Algorithms based on ICD‐10 criteria were used to derive binary variables. DAWBA‐based anxiety disorders included generalised anxiety disorder, separation anxiety disorder, specific phobias and social phobia at ages 7, 10 and 13 years (parent‐rated). These same disorders were available at ages 15 and 18 years (adolescent‐rated), except that participants were not asked about separation anxiety disorder but were asked about panic disorder and agoraphobia. Depressive diagnoses prior to age 13 were not used. Individuals with a diagnosis at any time point were classed as ‘diagnosis present’ and those without a diagnosis at all time points were classed as ‘diagnosis absent’.

### Polygenic risk scores

Polygenic risk scores (PRS) were derived using the standard method (The International Schizophrenia Consortium, 2009), based on a genome‐wide association study (GWAS) of 19,099 ADHD cases and 34,194 controls. See Data S1 for details. In brief, PRS were calculated for each individual by scoring the number of alleles (weighted by the allele effect size) across the ADHD discovery set of SNPs. PRS were standardised using z‐score transformations; odds ratios can be interpreted as increase in risk of the outcome, per standard deviation in PRS.

### Data analytic strategy

We first examined the overall association between ADHD PRS and presence of ADHD, anxiety and depression diagnoses. To test the first hypothesis that ADHD genetic risk is more strongly associated with anxiety or depression in females, we tested the association between ADHD PRS and sex in individuals with diagnosed anxiety, depression or either disorder. Assuming that individuals with comorbid diagnoses of ADHD and anxiety or depression have a more complex clinical presentation, we conducted sensitivity analyses excluding individuals with diagnosed ADHD (CATSS registry‐based diagnoses and ALSPAC algorithm‐based diagnoses). To test the second hypothesis, that females with ADHD have an increased burden of ADHD genetic risk, we examined the association between ADHD PRS and sex, in children with ADHD.

Analyses in CATSS were run using generalised estimating equations in R (*drgee* package), using family ID to cluster the data to account for related samples, adjusting for age. In ALSPAC, analyses were run using logistic regression models. Males were the reference sex, coded as ‘0’ and females were coded as ‘1’ for all analyses.

### Secondary analyses

The main analyses were repeated using ADHD PRS derived using alternative *p*‐value thresholds to examine the sensitivity of the results.

To determine whether putative diagnostic biases are associated with a delay in obtaining an ADHD diagnosis in females, we tested in CATSS whether age at first diagnosis (calculated as: [first_diagnosis_date – birth_year]/365.25) was associated with sex. We also tested whether including age at first diagnosis as a covariate affected the results.

To determine whether sex differences in severity of symptoms affected the registry‐based results in CATSS, analyses were adjusted for screening‐based measures of: (a) parent‐reported ADHD symptom severity and impairment, and (b) parent‐ and self‐reported anxiety and depression symptom severity.

## Results

The numbers of individuals with registry‐based (CATSS), screening‐based (CATSS) and algorithm‐based (ALSPAC) diagnoses of ADHD, anxiety and depression are shown in Table 1. ADHD PRS were associated with increased risk for these diagnostic categories, although the confidence intervals overlapped with 1 for the associations with clinical diagnoses of depression and screening‐based research diagnoses of anxiety and depression separately in CATSS (Table 1). Females were more likely to have anxiety and depression (OR: 1.51–3.14) and less likely to have ADHD (OR: 0.25–0.48) as compared to males (*p* < .002), for all definitions (Table S1).

**Table 1:** Association of ADHD PRS with diagnostic outcomes

Definition	Diagnosis	*N* affected/unaffected^a^	OR (CI)	*p*
Registry‐based clinical diagnoses (CATSS)	ADHD	443/13029	1.39 (1.26–1.54)	7.2E‐11
	Anxiety	265/13207	1.16 (1.02–1.32)	.020
	Depression	217/13247	1.11 (0.97–1.29)	.12
	Anxiety/depression	388/13078	1.16 (1.04–1.29)	.0062
Screening‐based research diagnoses (CATSS)	ADHD	1226/12228	1.25 (1.17–1.34)	2.8E‐11
	Anxiety	296/2375	1.13 (0.98–1.30)	.084
	Depression	312/2239	1.11 (0.98–1.25)	.099
	Anxiety/depression	470/2083	1.12 (1.01–1.25)	.031
Algorithm‐based research diagnoses (ALSPAC)	ADHD	199/2732	1.76 (1.51–2.05)	4.9E‐13
	Anxiety	483/1867	1.20 (1.08–1.33)	.00046
	Depression	352/2349	1.19 (1.06–1.33)	.0027
	Anxiety/depression	724/1728	1.17 (1.07–1.27)	.00063

CI: 95% confidence interval. Odds ratios refer to association of ADHD PRS with risk of each diagnosis. ADHD, Attention deficit hyperactivity disorder; ALSPAC, Avon Longitudinal Study of Parents and Children; CATSS, Child and Adolescent Twin Study in Sweden; PRS, polygenic risk scores.

a N.B. In ALSPAC, individuals were considered to be unaffected only if they did not meet criteria for diagnoses at all time points; Ns of unaffected individuals are low due to missing data across time points.

### ADHD genetic risk and sex, in those with anxiety or depression

Table 2 displays the results of association analyses between ADHD PRS and sex in individuals diagnosed with anxiety or depression. In CATSS, females with registry‐based clinical diagnoses had higher ADHD PRS than diagnosed males, though the confidence interval overlapped with 1 for depression. When anxiety and depression were defined using screening measures in each study, the estimates were closer to 1 in both CATSS and ALSPAC. When individuals with comorbid ADHD diagnoses were excluded, the effect sizes of the association between ADHD PRS and sex increased in CATSS individuals with registry‐based diagnoses of anxiety or depression (Table 2). This exclusion had no clear impact on analyses using research‐based diagnoses in either study.

**Table 2:** Association of ADHD PRS with sex of individuals with anxiety or depression diagnoses

Definition	Diagnosis	All diagnosed individuals			Excluding children with comorbid ADHD diagnosis		
		Males/Females	OR (CI)	*p*	Males/Females	OR (CI)	*p*
Registry‐based clinical diagnoses (CATSS)	Anxiety	107/158	1.42 (1.09–1.86)	.01	73/136	1.75 (1.26–2.44)	.00087
	Depression	79/138	1.35 (0.98–1.85)	.062	59/119	1.68 (1.15–2.47)	.0074
	Anxiety/depression	154/234	1.39 (1.12–1.73)	.0031	111/202	1.66 (1.28–2.16)	.00013
Screening‐based research diagnoses (CATSS)	Anxiety	65/231	1.08 (0.83–1.40)	.58	59/218	1.08 (0.82–1.41)	.60
	Depression	89/223	1.15 (0.88–1.50)	.32	77/209	1.15 (0.86–1.53)	.34
	Anxiety/depression	119/351	1.15 (0.94–1.42)	.18	107/334	1.15 (0.92–1.42)	.21
Algorithm‐based research diagnoses (ALSPAC)	Anxiety	178/305	1.07 (0.89–1.29)	.48	67/138	1.00 (0.75–1.35)	.99
	Depression	98/254	1.08 (0.87–1.36)	.48	46/121	1.08 (0.79–1.49)	.62
	Anxiety/depression	249/475	1.04 (0.89–1.21)	.65	101/221	1.02 (0.81–1.28)	.88

CI: 95% confidence interval. Males are the reference sex, coded as ‘0’ and females are coded as ‘1’. Odds ratios refer to association of ADHD PRS with being a female. ADHD, Attention deficit hyperactivity disorder; ALSPAC, Avon Longitudinal Study of Parents and Children; CATSS, Child and Adolescent Twin Study in Sweden; PRS, polygenic risk scores.

### ADHD genetic risk and sex, in those with ADHD

There was no statistically significant difference in ADHD PRS between females and males with ADHD, for any ADHD diagnostic definition (Table 3).

**Table 3:** Association of ADHD PRS with sex of individuals with ADHD diagnosis

Definition	Males/Females	OR (CI)	*p*
Registry‐based clinical diagnoses (CATSS)	312/131	1.04 (0.84–1.30)	.71
Screening‐based research diagnoses (CATSS)	809/417	0.96 (0.85–1.08)	.49
Algorithm‐based research diagnoses (ALSPAC)	157/42	1.15 (0.78–1.68)	.48

CI: 95% confidence interval. Males are the reference sex, coded as ‘0’ and females are coded as ‘1’. Odds ratios refer to association of ADHD PRS with being a female. ADHD, Attention deficit hyperactivity disorder; ALSPAC, Avon Longitudinal Study of Parents and Children; CATSS, Child and Adolescent Twin Study in Sweden; PRS, polygenic risk scores.

### Secondary analyses

Results using ADHD PRS derived based on different *p*‐value thresholds are shown in Figures S1–S3. The pattern of results was similar to the main results.

In CATSS, females were older than males at first registry‐based clinical diagnosis of ADHD [OR (CI) = 1.11 (1.02–1.20)], with somewhat wider confidence intervals for anxiety [OR (CI) = 1.14 (1.00–1.29)] and much wider confidence intervals for depression [OR (CI) = 0.93 (0.81–1.07)]. Including age at first diagnosis as a covariate did not substantially affect the results (Table S2).

In CATSS individuals with registry‐based diagnoses of anxiety or depression, males had more ADHD symptoms and were more likely to have impairment from ADHD symptoms, whereas females had more self‐reported anxiety and depressive symptoms at age 18, though parent‐reported symptoms did not differ by sex (Table S3). Adjusting the main analyses for ADHD severity and impairment continued to show a similar pattern of results, albeit with increased effect sizes (Table S4). Including symptom severity for anxiety and depression increased the effect size for the analysis of anxiety [OR (CI) = 2.94 (1.06–8.13)] but decreased the effect for depression [OR (CI) = 1.10 (0.62–1.96)] (Table S4).

## Discussion

The results of this study suggest that ADHD common genetic risk burden is higher in females with clinically recognised anxiety and depression than in affected males, but that this finding does not extend to diagnostic definitions based on whole sample research screening in the general population. No sex differences in ADHD genetic burden were found in individuals with ADHD based on clinical or research diagnostic definitions.

Consistent with studies showing genetic overlap across ADHD and other psychiatric phenotypes (e.g. Demontis et al., 2017), ADHD PRS were associated with increased risk of anxiety and depression in children from the general population, using a variety of diagnostic definitions. When comparing polygenic burden in females and males with clinical diagnoses of anxiety or depression based on the Swedish patient registry, females had higher ADHD PRS than males. Excluding individuals receiving a comorbid diagnosis of ADHD increased the strength of these associations. Increased effect sizes were also observed when ADHD symptom severity and impairment were included as covariates. One possible interpretation of these results is that females at genetic risk for ADHD may be underdiagnosed with ADHD and instead diagnosed with other psychiatric disorders such as anxiety or depression.

We did not see sex differences in ADHD PRS in those with anxiety or depression defined using research‐based diagnoses in either sample. However, several important differences between measures need to be noted in order to interpret this discrepancy. The research diagnoses in CATSS (screening‐based) and ALSPAC (algorithm‐based) are free from referral biases as they were obtained for all individuals (albeit subject to potentially nonrandom study attrition). In CATSS, high symptom levels reported by parents or adolescents at age 18 were used as diagnostic proxies. In ALSPAC, computer algorithms were applied to obtain a broad indicator of parent‐ or adolescent‐reported problems across ages 7–18 years. In contrast, Swedish registry‐based diagnoses are independent of the study protocol and liable to referral and diagnostic biases; they reflect ‘real‐life’ clinician's diagnoses, which are more sensitive to factors such as impairment and incorporate clinically relevant information from multiple informants. Despite these rather different diagnostic definitions, the sample sizes for comparisons within diagnostic groups were similar so differences in statistical power are unlikely to account for differences in effect sizes.

However, given these measurement differences, it is possible that the group of CATSS individuals who received registry‐based diagnoses are more severely affected compared to individuals meeting screening cutoffs. Indeed, CATSS individuals who screened positively for anxiety or depression at age 18 years and also had registry‐based diagnoses scored higher on parent‐ and self‐reported measures than those in the screened group who had not received a registry‐based diagnosis (see Table S5). This could suggest that there is no genetic difference in ADHD PRS by sex based on research diagnoses in CATSS and ALSPAC because sex differences are only present in more severely affected individuals.

Another possibility is that females who received registry‐based clinical diagnoses of anxiety or depression were more severely affected than males who received these diagnoses. In fact, the results suggest that in those with anxiety or depression, males had more ADHD symptoms and were more likely to have reported impairment than females; accounting for these variables increased the effect sizes, as discussed above. On the other hand, in this diagnosed group, females had higher levels of self‐reported anxiety and depression symptoms than males; including these variables in the models increased the effect size for the sex difference in ADHD PRS in those with anxiety but decreased this effect size in those with depression. This suggests that differences in symptom severity may account for the observed results for depression but not anxiety. A limitation of this analysis was the reduced sample size, which increased the confidence intervals. Also, information on impairment from anxiety and depressive symptoms was not available.

The results also showed that in individuals who received a registry‐based diagnosis of ADHD, females had a later age at first diagnosis than males. This could suggest that there is a delay in obtaining an ADHD diagnosis in females, which might be reflected in the more similar sex ratio of ADHD seen in adults (Faraone et al., 2015). However, it is also possible that there is a genuine sex difference in ADHD onset; future studies will need to examine this possibility.

Bearing in mind the measurement differences and secondary analyses discussed above, the results suggest that females at genetic risk for ADHD may be more likely to receive a clinical diagnosis of anxiety or depression than ADHD as compared to males. Given that there were no sex differences in ADHD genetic risk in affected individuals for the research‐based diagnostic definitions where all individuals were carefully screened for these disorders, this could suggest that referral or diagnostic biases contribute to the sex difference in prevalence of ADHD. Alternatively, genetic risk for ADHD may simply manifest differently in males and females, with females more likely to present with clinically relevant anxiety and depression rather than ADHD. We cannot distinguish between these two possibilities in this study. Either way, these results may partly explain the lower prevalence of ADHD seen in females in clinical samples, although not necessarily why mean differences in ADHD symptoms by sex are observed in population‐based samples.

We also examined the possibility of a female protective effect in ADHD. In contrast to previous studies (Hamshere et al., 2013; Martin et al., 2014), we did not see any evidence for females with ADHD (using several definitions) carrying a higher burden of ADHD common risk variants. Notably, one of these previous studies (Martin et al., 2014) also used ALSPAC data and found consistently higher ADHD PRS in females as compared to males, using a variety of definitions of ADHD problems. However, this previous study used a substantially smaller discovery dataset (727 cases and 5,081 controls) for calculating ADHD PRS, which may account for the inconsistent results in the current study. Our results are in line with a more recent, larger study that found no sex difference in ADHD PRS in ADHD cases (Martin et al., 2017). Although several family studies support the female protective effect hypothesis by finding a modest increased risk of ADHD in first‐degree relatives of affected females, compared to relatives of affected males (Martin et al., 2017; Rhee & Waldman, 2004; Smalley et al., 2000; Taylor et al., 2016), there appears to be no strong or consistent sex difference in ADHD polygenic burden.

The main strengths of this study rely on utilising the largest available ADHD GWAS to calculate PRS in two well‐characterised population‐based samples, with several different (clinical and research) diagnostic definitions. However, the target sample sizes of those with psychiatric diagnoses were low, limiting statistical power, and the effect sizes were modest, though typical for PRS studies of psychiatric phenotypes (e.g. Demontis et al., 2017; Hamshere et al., 2013; Martin et al., 2014). Also, as with all analyses of longitudinal datasets, the study is limited by possible attrition biases. Previous work suggests that ALSPAC individuals with missing data have a higher burden of genetic risk for schizophrenia, although no sex bias was observed in availability of genetic data (Martin et al., 2016). However, we cannot entirely rule out the possibility that selective attrition of those with more severe phenotypes or higher genetic risk for ADHD biased our analyses of research‐based diagnoses. As the focus of the study was on common autosomal variants, we cannot extrapolate our findings to rare and sex chromosomal variants.

## Conclusion

This study provides genetic evidence that ADHD risk may be more likely to manifest as anxiety or depression in referred females than in males and may partly explain the lower prevalence of diagnosed ADHD in females. Replication of these findings in other population genetic datasets that have study‐independent information for clinical diagnoses is needed to confirm these results. This study suggests that females presenting with clinical symptoms of anxiety or depression might benefit from additional careful screening for ADHD, particularly if they have a family history of ADHD.

**Key points**

- ADHD is more commonly diagnosed in males than females in clinical and population samples.
- This general population study suggests that ADHD genetic risk might more commonly manifest as anxiety or depression in females than in males.
- The results do not support an increased burden of common genetic risk variants in females with ADHD.
- Females at familial risk for ADHD, with anxiety and depression problems would benefit from careful screening for ADHD.

## Supporting information


**Data S1.** Polygenic risk score calculation.
**Table S1**. Association between ADHD, anxiety and depression diagnoses with sex.
**Table S2**. Association of ADHD PRS with sex of individuals with ADHD, anxiety and depression registry‐based diagnoses in CATSS, including age at first diagnosis as a covariate.
**Table S3**. Association between variables related to severity of clinical presentation and sex.
**Table S4**. Association of ADHD PRS with sex of CATSS individuals with registry‐based diagnoses of anxiety or depression, after adjusting for severity of clinical presentation.
**Table S5**. Mean symptoms in CATSS individuals screening positively for anxiety or depression, depending on whether they also received registry‐based diagnoses.
**Figure S1**. Variance explained in diagnostic outcomes by ADHD PRS derived using variable *p*‐value thresholds.
**Figure S2**. Variance explained in sex by ADHD PRS derived using variable *p*‐value thresholds, in individuals with diagnostic outcomes.
**Figure S3**. Variance explained in sex by ADHD PRS derived using variable *p*‐value thresholds, in individuals with diagnostic outcomes, excluding individuals with ADHD.
