# Supplementary material for: Sex‐specific manifestation of genetic risk for attention deficit hyperactivity disorder in the general population
Source: J Child Psychol Psychiatry. 2018 Feb 16;59(8):908–16. doi: 10.1111/jcpp.12874 (PMC6055636; doi:10.1111/jcpp.12874)
Supplement: Supplementary file 1 — Data S1. Polygenic risk score calculation. Table S1. Association between ADHD, anxiety and depression diagnoses with sex. Table S2. Association of ADHD PRS with sex of individuals with ADHD, anxiety and depression registry‐based diagnoses in CATSS, including age at first diagnosis as a covariate. Table S3. Association between variables related to severity of clinical presentation and sex. Table S4. Association of ADHD PRS with sex of CATSS individuals with registry‐based diagnoses of anxiety or depression, after adjusting for severity of clinical presentation. Table S5. Mean symptoms in CATSS individuals screening positively for anxiety or depression, depending on whether they also received registry‐based diagnoses. Figure S1. Variance explained in diagnostic outcomes by ADHD PRS derived using variable p‐value thresholds. Figure S2. Variance explained in sex by ADHD PRS derived using variable p‐value thresholds, in individuals with diagnostic outcomes. Figure S3. Variance explained in sex by ADHD PRS derived using variable p‐value thresholds, in individuals with diagnostic outcomes, excluding individuals with ADHD. [file JCPP-59-908-s001.docx]

# Online Supporting Information for: Sex-specific manifestation of genetic risk for ADHD in the general population – by Martin et al.

## Polygenic risk score calculation

Polygenic risk scores (PRS) were derived with PLINK.v.1.9, based on a genome-wide association study (GWAS) meta-analysis of 20,183 ADHD cases and 35,191 controls (in CATSS, the meta-analysis of 19,099 ADHD cases and 34,194 controls of European ancestry was used). The results of this GWAS were filtered based on imputation quality (INFO>0.8) and minor allele frequency (MAF>0.05). Indels, multi-allelic and asymmetric/ambiguous (AT, TA, CG, GC; CATSS-only) SNPs were excluded. LD-clumping was run to obtain a relatively independent set of SNPs, while retaining the most significant SNP in each LD (linkage disequilibrium) block. The following parameters were applied in PLINK: --clump-kb 1000 --clump-r2 0.1.

The total number of independent SNPs used to derive the PRS was N=84,969 in CATSS and N=78,781 in ALSPAC. PRS were derived using a variety of p-value thresholds to select SNPs (p<1, p<0.5, p<0.1, p<0.05, p<0.01). The primary analyses used PRS derived for SNPs with p<0.1, which were based on N=26,542 SNPs in CATSS and N=23,968 in ALSPAC. PRS were calculated for each individual in the CATSS and ALSPAC imputed data by scoring the number of alleles (weighted by the SNP log of the odds ratio) across the set of SNPs in PLINK (using the command --score). PRS were standardised using z-score transformations; odds ratios can be interpreted as increase in risk of the outcome, per standard deviation in PRS. Nagelkerke R^2^ differences between null and full models were calculated to obtain estimates of variance explained.

Study-specific principal components (PCs) were included as covariates to account for population stratification. In CATSS, the results of a principal components analysis in PLINK (after LD-pruning and removing SNPs located in long-range LD regions) were used to obtain PCs. PCs were calculated on unrelated individuals and projected onto relatives. The ALSPAC team used EIGENSTRAT to generate the top PCs after removal of known long LD regions. The first 5 PCs for each study were included as covariates in all analyses.

## Table S1: Association between ADHD, anxiety and depression diagnoses with sex

| Definition | Outcome | OR(CI) | p |
| --- | --- | --- | --- |
| Registry-based clinical diagnoses (CATSS) | ADHD | 0.41(0.33-0.51) | 2.3E-15 |
|  | Anxiety | 1.51(1.16-1.95) | 0.002 |
|  | Depression | 1.79(1.34-2.40) | 8.6E-05 |
|  | Anxiety or depression | 1.56(1.26-1.94) | 6.0E-05 |
| Screening-based research diagnoses (CATSS) | ADHD | 0.48(0.42-0.55) | 4.9E-28 |
|  | Anxiety | 3.14(2.33-4.25) | 1.0E-13 |
|  | Depression | 2.17(1.65-2.85) | 3.2E-08 |
|  | Anxiety or depression | 2.70(2.14-3.42) | 1.4E-16 |
| Algorithm-based research diagnoses (ALSPAC) | ADHD | 0.25(0.17-0.35) | 3.7E-15 |
|  | Anxiety | 1.51(1.23-1.85) | 9.3E-05 |
|  | Depression | 2.26(1.77-2.90) | 8.7E-11 |
|  | Anxiety or depression | 1.75(1.46-2.10) | 1.1E-09 |

CI: 95% confidence interval. Males are the reference sex, coded as ‘0’ and females are coded as ‘1’. Odds ratios refer to association of each diagnosis with being a female.

## Table S2: Association of ADHD PRS with sex of individuals with ADHD, anxiety and depression registry-based diagnoses in CATSS, including age at first diagnosis as a covariate

| Outcome | Males | Females | OR(CI) | p |
| --- | --- | --- | --- | --- |
| ADHD | 312 | 131 | 1.03(0.83-1.28) | 0.79 |
| Anxiety | 107 | 158 | 1.40(1.07-1.84) | 0.014 |
| Depression | 79 | 138 | 1.34(0.98-1.83) | 0.068 |
| Anxiety* | 73 | 136 | 1.73(1.24-2.41) | 0.0013 |
| Depression* | 59 | 119 | 1.67(1.14-2.45) | 0.0085 |

* Secondary analyses, excluding children with ADHD diagnoses. Males are the reference sex, coded as ‘0’ and females are coded as ‘1’. Odds ratios refer to association of ADHD PRS with being a female.

## Table S3: Association between variables related to severity of clinical presentation and sex

| Variable | Anxiety | | | Depression | | | Anxiety or Depression | | |
| --- | --- | --- | --- | --- | --- | --- | --- | --- | --- |
|  | **N** | **OR(CI)** | **p** | **N** | **OR(CI)** | **p** | **N** | **OR(CI)** | **p** |
| ADHD symptoms | 265 | 0.92(0.85-0.98) | 0.015 | 217 | 0.90 (0.82-0.98) | 0.018 | 388 | 0.90 (0.85-0.96) | 0.00094 |
| ADHD impairment | 265 | 0.46(0.24-0.89) | 0.02 | 217 | 0.37 (0.18-0.77) | 0.0083 | 388 | 0.39 (0.23-0.66) | 0.00055 |
| Anxiety parent-rated symptoms | 107 | 1.02(0.89-1.18) | 0.74 | 100 | 1.02 (0.87-1.20) | 0.81 | 158 | 1.06(0.94-1.21) | 0.35 |
| Depression parent-rated symptoms | 108 | 1.02(0.96-1.08) | 0.61 | 99 | 1.05 (0.98-1.12) | 0.16 | 159 | 1.03(0.97-1.09) | 0.30 |
| Anxiety self-rated symptoms | 106 | 1.05(1.01-1.10) | 0.01 | 95 | 1.05 (1.01-1.09) | 0.015 | 161 | 1.05(1.02-1.09) | 0.0017 |
| Depression self-rated symptoms | 107 | 1.09(1.01-1.17) | 0.021 | 93 | 1.06 (0.99-1.14) | 0.12 | 160 | 1.07(1.01-1.13) | 0.018 |

CI: 95% confidence interval. Males are the reference sex, coded as ‘0’ and females are coded as ‘1’. Odds ratios refer to association of clinical symptoms with being a female.

## Table S4: Association of ADHD PRS with sex of CATSS individuals with registry-based diagnoses of anxiety or depression, after adjusting for severity of clinical presentation

| Diagnosis | Adjusting for ADHD | | | | Adjusting for anxiety/depression | | | |
| --- | --- | --- | --- | --- | --- | --- | --- | --- |
|  | **M** | **F** | **OR(CI)** | **p** | **M** | **F** | **OR(CI)** | **p** |
| Anxiety | 107 | 158 | 1.51(1.14-2.00) | 0.0041 | 19 | 57 | 2.94(1.06-8.13) | 0.038 |
| Depression | 79 | 138 | 1.45(1.04-2.01) | 0.028 | 15 | 54 | 1.10(0.62-1.96) | 0.74 |
| Anxiety/ depression | 154 | 234 | 1.48(1.18-1.86) | 0.00072 | 28 | 88 | 1.49(0.91-2.43) | 0.11 |

CI: 95% confidence interval. M: males; F: females. Males are the reference sex, coded as ‘0’ and females are coded as ‘1’. Odds ratios refer to association of ADHD PRS with being a female.

## Table S5: Mean symptoms in CATSS individuals screening positively for anxiety or depression, depending on whether they also received registry-based diagnoses

| Variable | Screening-only (N=367) | Screening+registry (N=103) | OR(CI) | p |
| --- | --- | --- | --- | --- |
|  | **Mean(SD)** | **Mean(SD)** |  |  |
| Anxiety parent-rated symptoms | 3.4(2.5) | 5.1(3.1) | 1.25(1.13-1.39) | 1.20E-05 |
| Depression parent-rated symptoms | 5.4(4.4) | 9.8(6.2) | 1.18(1.12-1.25) | 3.10E-09 |
| Anxiety self-rated symptoms | 28.1(12.9) | 31.7(14.2) | 1.02(1.00-1.04) | 0.034 |
| Depression self-rated symptoms | 15.7(6.8) | 17.8(7.1) | 1.05(1.00-1.09) | 0.035 |

CI: 95% confidence interval. Screening-only group are the reference, coded as ‘0’ and individuals with both screening- and registry-based diagnoses are coded as ‘1’. Odds ratios refer to association of clinical symptoms with meeting both screening- and registry-based diagnoses.

## Figure S1: Variance explained in diagnostic outcomes by ADHD PRS derived using variable p-value thresholds


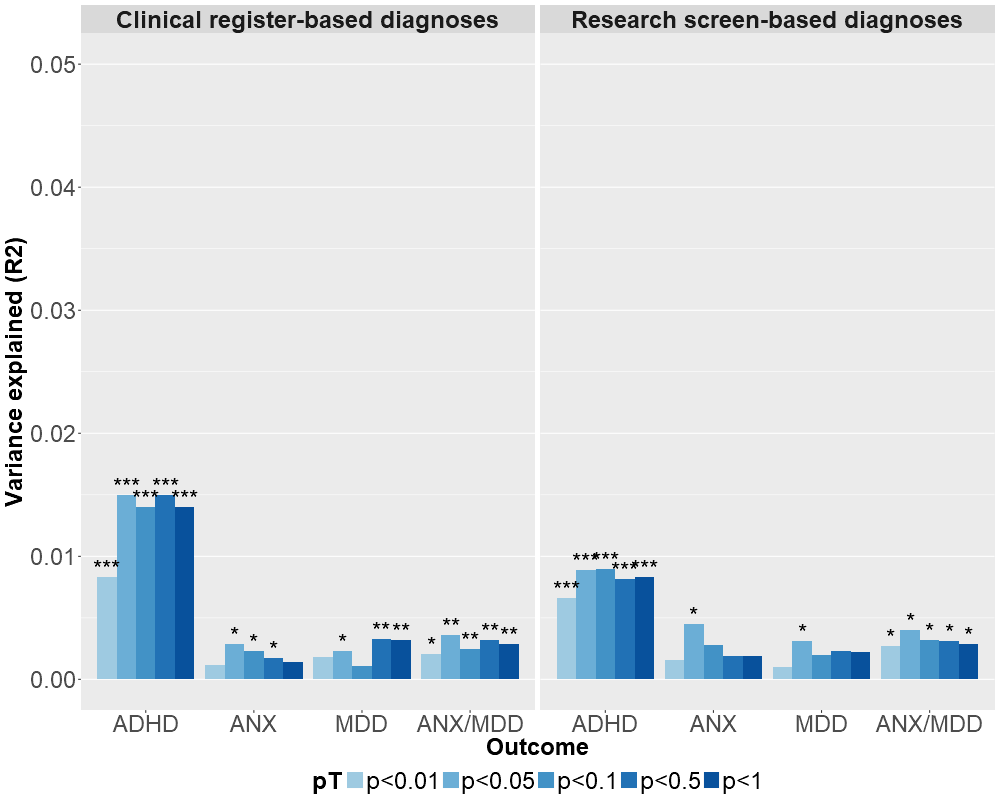

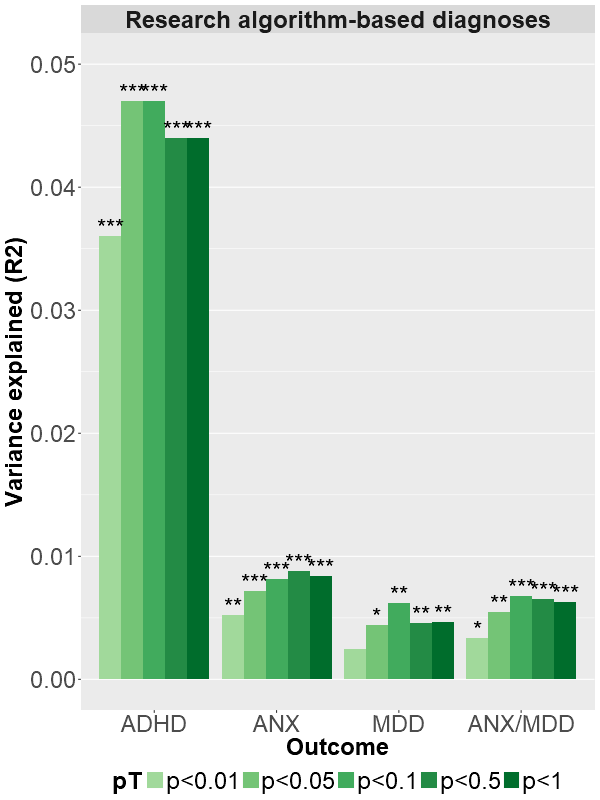


**Fig. S1:** Results in CATSS (left) and ALSPAC (right). N.B. The amounts of variance explained correspond to Nagelkerke R^2^ differences between null and full models and are affected by differential case-control ratios for different diagnostic definitions; in particular, the Ns of control individuals in ALSPAC are low due to missing data across time points as individuals were only considered to be unaffected if they did not meet criteria for diagnoses at any time point. ADHD: Attention deficit hyperactivity disorder; ANX: any anxiety disorder; MDD: any major depressive disorder.

## Figure S2: Variance explained in sex by ADHD PRS derived using variable p-value thresholds, in individuals with diagnostic outcomes


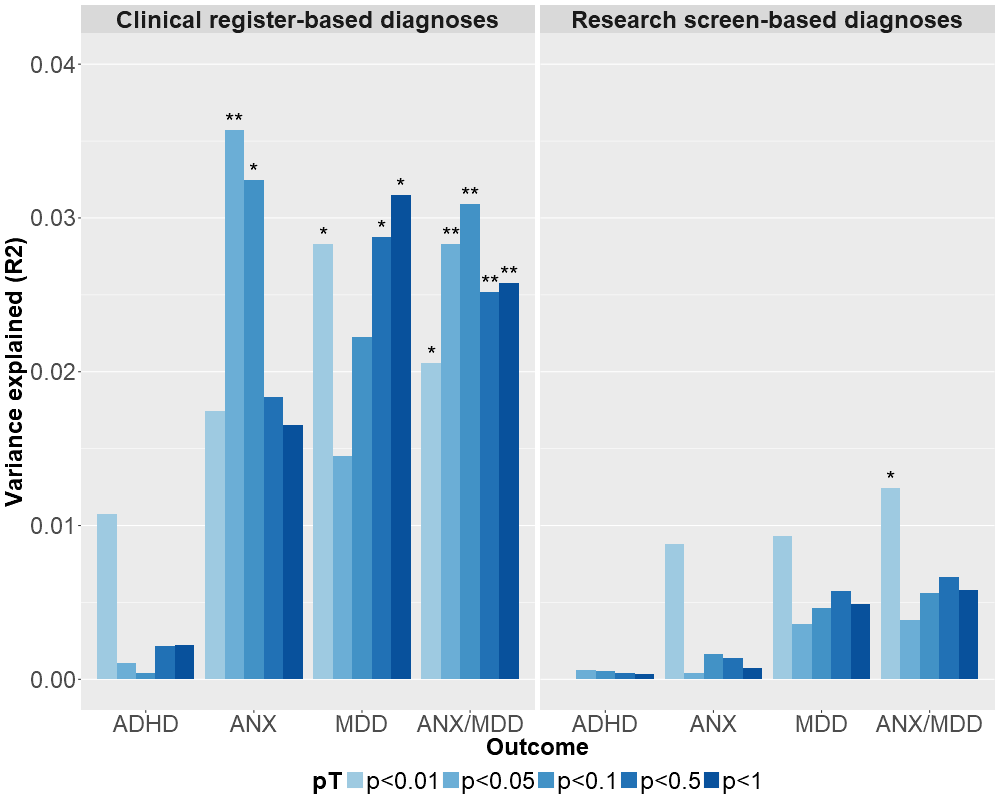

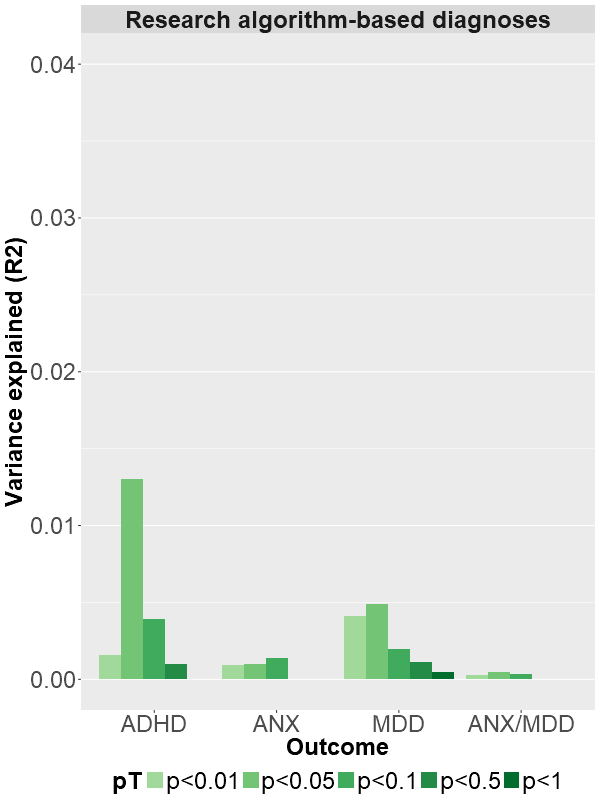


**Fig. S2:** Results in CATSS (left) and ALSPAC (right). N.B. The amounts of variance explained correspond to Nagelkerke R^2^ differences between null and full models and are affected by differential male-female ratios for different diagnostic definitions. ADHD: Attention deficit hyperactivity disorder; ANX: any anxiety disorder; MDD: any major depressive disorder.

## Figure S3: Variance explained in sex by ADHD PRS derived using variable p-value thresholds, in individuals with diagnostic outcomes, excluding individuals with ADHD


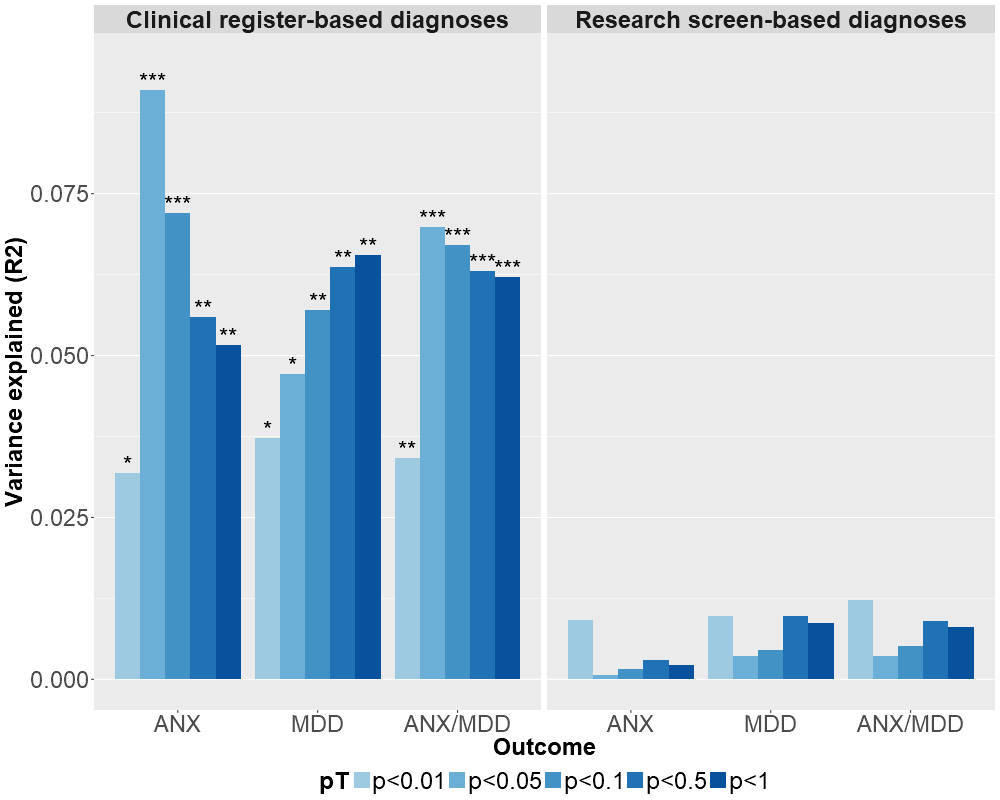

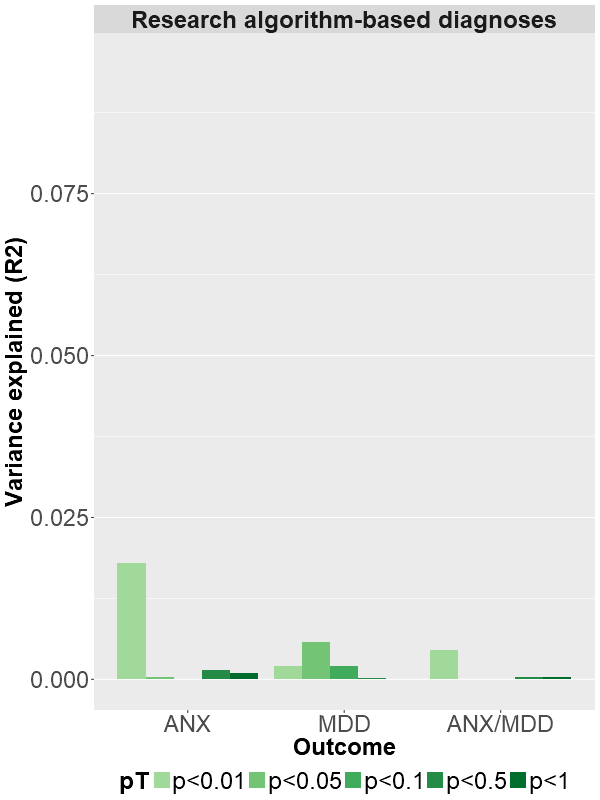


**Fig. S3:** Results in CATSS (left) and ALSPAC (right). N.B. The amounts of variance explained correspond to Nagelkerke R^2^ differences between null and full models and are affected by differential male-female ratios for different diagnostic definitions. ADHD: Attention deficit hyperactivity disorder; ANX: any anxiety disorder; MDD: any major depressive disorder.
